# Death and beauty: mortality salience and creatureliness increase self-objectification not only in females but also in males

**DOI:** 10.3389/fpsyg.2025.1512704

**Published:** 2025-02-20

**Authors:** Yang Gao, Kexin Lu, Yichen Ni, Yang Shen

**Affiliations:** ^1^School of Public Management, Northwest University, Xi’an, China; ^2^Mental Health Education and Counseling Center of Tongji University, Shanghai, China; ^3^Department of Senior High School, Shenzhen Foreign Language School, Shenzhen, Guangdong, China; ^4^Collaborative Innovation Center of Assessment for Basic Education Quality, Beijing Normal University, Beijing, China

**Keywords:** mortality salience, self-objectification, TMT, death anxiety, cultural worldview, creatureliness

## Abstract

**Background:**

Self-objectification, the tendency to perceive oneself as an object subject to external evaluation, negatively impacts wellbeing, contributing to issues such as anxiety and eating disorders. While objectification theory outlines its societal underpinnings, it provides limited insight into the psychological mechanisms that sustain its prevalence. Terror Management Theory (TMT) posits that self-objectification functions as a defense against death anxiety, operating through two pathways: cultural worldview compliance (adherence to objectifying societal norms) and suppressing the awareness of creatureliness (avoiding awareness of humans’ biological vulnerability and animalistic nature). This research explores these mechanisms and their gender-specific dynamics under mortality salience (MS).

**Methods:**

This study includes three experimental studies. The study 1 examined baseline gender differences in perceived creatureliness and adherence to objectification culture. Study 2 used a 2 (MS/control) × 2 (gender: male/female) design to investigate the effects of MS and gender on self-objectification with cultural worldview compliance as a continuous moderator. Study 3 employed a 2 (MS/control) × 2 (creatureliness: heightened/reduced) × 2 (gender: male/female) design to assess the effects of creatureliness salience on self-objectification.

**Results:**

Study 1 revealed that women were more culturally objectified, whereas men exhibited higher perceived creatureliness. However, Study 2 and Study 3 found no significant gender-related interactions in self-objectification. Study 2 showed that MS increased self-objectification across genders, with women displaying higher self-objectification due to stronger adherence to objectification cultural norms. Study 3 demonstrated that heightened creatureliness salience amplified self-objectification under MS for both genders, highlighting the universal role of creatureliness suppression in existential defenses.

**Conclusion:**

These findings provide evidence for dual pathways—cultural worldview compliance and creatureliness suppression—underlying self-objectification as a defense against death anxiety. However, while cultural compliance explains gender differences in self-objectification at baseline, creatureliness suppression appears to function universally across genders. This study clarifies the boundaries of gender differences, emphasizing that the observed gender differences were limited to perceptions of objectification and creatureliness, rather than self-objectification itself. These insights contribute to interventions targeting the maladaptive effects of self-objectification, advocating for gender-sensitive approaches to enhance psychological wellbeing.

## 1 Introduction

Self-objectification, a widespread phenomenon with profound implications for mental health, is particularly pervasive in cultural contexts that emphasize the aesthetic value of women’s bodies (Jones and Griffiths, 2015). Defined as reducing individuals to their appearance, objectification involves treating a person—or their body parts—as an object for evaluation or gaze (Nussbaum, 1995). According to objectification theory, cultures saturated with appearance-based standards foster self-objectification, where individuals internalize external evaluations and view themselves from an observer’s perspective (Fredrickson and Roberts, 1997). Self-objectification is linked to adverse mental health outcomes, such as body image dissatisfaction, anxiety, and eating disorders (Calogero et al., 2011; Tiggemann and Williams, 2012). While objectification theory provides a robust framework for understanding the societal pressures that perpetuate self-objectification, it offers limited insight into the underlying psychological mechanisms that sustain this phenomenon across time and cultures (Moradi and Huang, 2008).

Terror Management Theory (TMT) offers a compelling perspective to address this gap, positing that adherence to the objectification culture functions as a mechanism for managing death anxiety (Goldenberg, 2013). TMT suggests that existential anxiety—arising from the awareness of mortality—is mitigated through alignment with cultural worldviews that provide symbolic immortality and meaning (Greenberg et al., 2003). Objectification culture serves dual psychological purposes: reinforcing cultural standards as a buffer against death anxiety (cultural worldview defense) (Goldenberg et al., 2005) and distancing individuals from reminders of their creatureliness, which is closely associated with mortality (Goldenberg et al., 2011). Existing research highlights how conforming to objectifying standards supports cultural worldview defense by reinforcing one’s value within a meaningful system (Goldenberg et al., 2000; Cox et al., 2009).

Furthermore, TMT attempts to provide an ultimate explanation for the origins of the cultural worldview of females rather than males, objectification, and why such cultural worldviews exist universally across different times and cultures (Goldenberg and Roberts, 2004; Grabe et al., 2005). Women’s bodies, often culturally linked to biological processes such as reproduction, may serve as potent reminders of creatureliness and mortality. In this context, “creatureliness suppression” refers to the cognitive and emotional strategies aimed at reducing the salience of mortality-related thoughts associated with biological characteristics. Specifically, objectifying women helps suppress the awareness of their animal nature by reducing them to non-living objects, thereby mitigating the mortality salience triggered by their creatureliness (Roberts et al., 2002; Goldenberg et al., 2013; Goldenberg et al., 2007). However, this hypothesis lacks robust empirical support. Furthermore, the rising objectification of men, particularly within sexual minority groups (Szymanski et al., 2019; Duggan and McCreary, 2004), challenges the creatureliness suppression perspective, as male bodies are not traditionally associated with mortality-linked biological processes. These gaps underscore the need to clarify whether self-objectification’s psychological functions—cultural worldview defense and creatureliness suppression —vary across genders and adapt to shifting cultural norms.

This study seeks to clarify the psychological mechanisms underlying self-objectification by integrating TMT’s dual function of cultural worldview defense and creatureliness suppression. Specifically, we aim to examine whether women’s greater susceptibility to self-objectification stems from their heightened internalization of objectifying cultural standards or suppression of the creatureliness associated with their biological characteristics. Furthermore, we explore whether mortality salience influences self-objectification in men, who traditionally experience less societal pressure to conform to appearance-based standards. By disentangling these pathways, this study extends TMT’s explanatory power, highlights the gendered nature of objectification culture, and provides insights into its persistence across societies. Ultimately, these findings aim to inform interventions that mitigate the adverse effects of self-objectification by addressing its underlying existential roots.

### 1.1 Cultural worldview defense function of (self-)objectification

Cultural worldviews help people cope with the fear of death by providing a shared understanding of reality that gives life meaning, order, and a sense of permanence (Becker, 1997). These worldviews promise immortality in two ways: symbolically, through lasting contributions to cultural values and norms, or literally, through beliefs in an afterlife or spiritual salvation (Greenberg et al., 1997). By following these shared cultural beliefs, individuals can reduce the anxiety of knowing their lives are finite (Lu et al., 2019). Research has consistently shown that when people are reminded of their mortality—a phenomenon called mortality salience—they tend to act in ways that align with their cultural values, a response known as cultural worldview defense (Solomon et al., 2004; Burke et al., 2010).

From a Terror Management Theory (TMT) perspective, the widespread objectification of women is one such cultural norm that helps people manage their fear of death (Goldenberg, 2013). Objectification reduces women’s bodies to objects of evaluation, turning them into symbols within a more extensive cultural system that offers stability and meaning. By adhering to these objectifying norms, individuals—both men and women—find reassurance in their connection to shared cultural values, which helps to reduce death-related fears (Cox et al., 2009; Arndt et al., 2003). Studies have shown that reminders of death can increase the tendency to objectify women, as conforming to these cultural norms helps reduce anxiety. For example, Grabe et al. found that both men and women were more likely to objectify women after being reminded of their mortality (Grabe et al., 2005). For women, these cultural norms often lead to self-objectification, where a person’s sense of self-worth becomes tied to their physical appearance (Siegel and Calogero, 2019). This self-focus not only aligns with societal standards but also serves as a way to cope with existential fears by boosting self-esteem (Goldenberg et al., 2005; Hart et al., 2005; Greenberg et al., 1986).

In summary, objectification and self-objectification serve as a cultural worldview defense and self-esteem defense to existential anxiety. These mechanisms explain why objectification culture persists, even though it negatively affects women’s physical and mental health. However, it does not fully address why the objectification of women is so widespread across different times and cultures. TMT offers a deeper insight, suggesting that the objectification of women helps people suppress the mortality salience triggered by women’s creatureliness (Goldenberg, 2013).

### 1.2 Creatureliness suppression function of objectification

Despite relying on cultural worldviews to mitigate death anxiety, the human body continually reminds us of our inherent vulnerability and inevitable mortality (Goldenberg et al., 2000; Goldenberg et al., 2006). The similarity between human and non-human (animal) characteristics evokes awareness about the body, highlighting our inherent mortality. In particular, women’s biological characteristics—especially those related to reproduction—are more pronounced than men’s, making their bodies more closely associated with animalistic and natural elements (Ortner, 1972; Reynolds and Haslam, 2011). The awareness of this connection between humans and animals triggers the need to deny or suppress these “creaturely” reminders, a psychological defense mechanism elaborated in TMT (Mountain and Marino, 2015; Beatson and Halloran, 2007; Cox et al., 2007).

Objectification provides a powerful mechanism for managing these existential threats by transforming women’s bodies into symbolic forms (Goldenberg, 2005; Goldenberg et al., 2007). This process distances individuals from mortality reminders by “de-creaturing” women’s physicality—removing its biological and creaturely connotations (Grabe et al., 2005; Morris et al., 2018). For example, after MS, men tend to evaluate objectifying advertisements (e.g., alcohol bottles shaped like women’s bodies) more favorably and find plastic mannequins more attractive than real women’s bodies (Goldenberg and Morris, 2016; Christina et al., 2017; Morris and Goldenberg, 2015). Similarly, MS leads women to self-objectify, focusing on their appearance and detaching from the biological aspects of their identity (Goldenberg et al., 2011; Heflick and Goldenberg, 2014), serving as a psychological coping mechanism. Together, these mechanisms explain why objectification remains such a persistent cultural phenomenon despite its harmful effects on women’s wellbeing (Fredrickson and Roberts, 1997; Hong et al., 2015).

### 1.3 Confusion between two pathways and ambiguity in the mechanisms of objectification

Although Terror Management Theory (TMT) offers valuable insights into the psychological mechanisms behind objectification and self-objectification, several critical assumptions still need to be explored. A key proposition of TMT is that women’s biological characteristics—particularly those tied to reproduction—evoke existential anxiety by reminding individuals of human creatureliness and mortality (Goldenberg, 2013). However, the empirical foundation for this assumption is limited, raising questions about its validity and broader implications.

#### 1.3.1 The ambiguity of female creatureliness as a mortality cue

TMT posits that women’s bodies serve as more salient reminders of mortality than men’s due to their pronounced biological roles in reproduction (Goldenberg, 2013). However, this assumption has yet to be conclusively tested. While women’s biological functions, such as menstruation and breastfeeding, are indeed visible markers of corporeality (Eisler, 1995), male bodies also exhibit traits tied to creatureliness, such as body hair, sweat, and aging-related decline (Goldenberg et al., 2000; Goldenberg, 2005). Unlike reproductive characteristics, these features are persistent rather than situational, suggesting that the salience of female creatureliness as a mortality cue may be context-dependent. Moreover, anthropological and historical perspectives complicate this narrative by revealing that many cultures have revered women’s bodies as symbols of life, fertility, and immortality (Yang et al., 2005). This dual symbolism—linking women’s bodies both to life and mortality—introduces a layer of ambiguity that existing research has yet to address fully.

#### 1.3.2 Methodological bias in research on creatureliness suppression

Empirical studies exploring the role of female creatureliness in objectification manipulate mortality salience (MS) by presenting images or scenarios involving women engaged in biological functions, such as pregnancy or breastfeeding (Cox et al., 2007; Goldenberg et al., 2007). These studies consistently show that MS increases both objectification and self-objectification of women. However, by focusing almost exclusively on female-specific features, this research neglects the potential for male creatureliness—such as the body, its functions, and its byproducts (Goldenberg et al., 2000; Goldenberg et al., 2006; Goldenberg, 2005)—to function similarly. This methodological bias raises a crucial question: are the observed effects on female objectification due to their heightened creatureliness, or are they simply a result of the research emphasis on women’s biological characteristics? Addressing this gap requires experimental designs incorporating male or non-gendered creatureliness and examining how MS influences objectification across genders.

#### 1.3.3 Growing cultural norms around male objectification

Although cultural norms around objectifying men are less pervasive than those around women, they are increasingly evident. Media portrayals of idealized male bodies, emphasizing muscularity and youthfulness, have been linked to body dissatisfaction, reduced self-esteem, and unhealthy behaviors such as excessive exercise or steroid use (Thornborrow et al., 2020; Grabe et al., 2008). While these norms suggest that men are not immune to objectification, they differ significantly from those applied to women. For women, physical appearance is often the primary determinant of social value, while for men, it is secondary to other dimensions, such as professional success or financial status (Moradi, 2010; Fredrickson and Roberts, 1997). These differences warrant further empirical scrutiny, particularly to examine whether male objectification serves similar psychological functions under MS.

#### 1.3.4 The overlap between creatureliness suppression and cultural worldview defense

The methods used to activate awareness of female creatureliness often intersect with cultural taboos surrounding women’s bodies (Hebl et al., 2004; Johnston-Robledo et al., 2007), further complicating the interpretation of findings on objectification under mortality salience (MS). For example, menstruation and breastfeeding are frequently stigmatized, reflecting societal discomfort with female physiology (Heine et al., 2006). Empirical studies demonstrate that exposing participants to these taboo subjects under MS can elicit adverse reactions that go beyond avoiding reminders of creatureliness (Cox et al., 2007; Goldenberg and Roberts, 2004). For instance, Roberts et al. found that when a woman dropped a tampon in public, participants reinforced cultural norms by intensifying their evaluations of her physical appearance (Roberts et al., 2002). Such findings suggest that objectification may not solely serve as a defense against creatureliness but also as a mechanism to reassert cultural values when existing norms are challenged (Rosenblatt et al., 1989; Wollast et al., 2021).

This overlap between creatureliness suppression and cultural worldview defense introduces ambiguity in interpreting the mechanisms underlying objectification under MS. Are individuals primarily motivated to avoid reminders of mortality by symbolically de-creaturing women’s bodies (Morris et al., 2018), or does compliance with cultural norms of objectification indirectly mitigate creatureliness? This “chicken-and-egg” dilemma underscores the need for research that disentangles these pathways. For example, experimental designs that make general human creatureliness (e.g., the body, body functions) salient—rather than focusing exclusively on female-specific features—could help clarify the role of creatureliness suppression in objectification.

Finally, most research on objectification and creatureliness suppression has been conducted in Western contexts. Non-Western societies may emphasize different attributes, such as collectivism or holistic views of the body (Thornborrow et al., 2020; Xu et al., 2010), which could alter the salience of creatureliness and the dynamics of objectification (Merino et al., 2024).

### 1.4 The present research and hypotheses

This research aims to disentangle the dual pathways—cultural worldview compliance and creatureliness suppression—through which mortality salience (MS) may drive self-objectification. We focus on clarifying the gendered nature of these mechanisms to address longstanding questions about the role of women’s biological characteristics, the universality of creatureliness suppression, and whether cultural objectification norms operate similarly across genders. By doing so, we seek to determine whether women’s prominence in objectification culture is a byproduct of their more salient biology or if cultural worldviews and creatureliness suppression function independently of gendered assumptions. Ultimately, these findings will inform strategies to mitigate the harmful mental health consequences of objectification and enhance wellbeing across genders in an increasingly image-conscious world (Schaefer et al., 2017; Merino et al., 2024).

Previous literature has assumed that women’s bodies are more strongly evoke mortality-related anxieties (Goldenberg et al., 2013), yet direct comparisons of men’s and women’s creatureliness under parallel conditions are lacking. Similarly, while it is well-documented that women tend to self-objectify more due to cultural norms emphasizing their appearance (Fredrickson and Roberts, 1997), it remains to be seen whether this difference persists even when both genders’ biological vulnerabilities are highlighted equally. We hypothesize that when biological features are made equally salient for both genders, men and women will not differ significantly in perceived creatureliness (H1). The notion that women inherently represent greater creatureliness than men will not hold when both are viewed under comparable biological conditions. Despite comparable creatureliness, women will report higher tendencies toward self-objectification at baseline, reflecting the more decisive influence of objectifying cultural norms on female self-perception (H2).

Study 2 focuses on the cultural worldview compliance pathway. Given that objectification culture is deeply ingrained—especially for women who face more intense pressures to conform to appearance standards (Hebl et al., 2004; Moradi and Huang, 2008)—we expect MS to heighten self-objectification across genders, but more strongly in women due to their internalized cultural norms. We hypothesize that under MS, both men and women will show increased self-objectification. However, this effect will be more substantial among women, reflecting their deeper internalization of objectifying cultural worldviews (H3). Adherence to objectification cultural norms will moderate the MS–self–objectification relationship in both genders. Individuals who strongly endorse objectifying norms will exhibit a more significant increase in self-objectification after MS, indicating that cultural worldview compliance is a key mechanism in how mortality concerns translate into self-objectifying behavior (H4).

Study 3 shifts the focus to the creatureliness suppression pathway, investigating whether highlighting human-animal similarity amplifies the effects of MS on self-objectification. If objectification functions to deny our biological roots and thus dampen death anxiety, making creatureliness salient should intensify self-objectification for both genders. We hypothesize that under MS combined with the heightened awareness of human creatureliness (applied equally to both genders), self-objectification will increase in both men and women, demonstrating that creatureliness suppression—like cultural worldview compliance—is not inherently gender-specific (H5). Furthermore, the internalization of objectifying cultural norms will mediate the relationship between MS and self-objectification when creatureliness is made salient. In other words, acknowledging our shared animalistic nature intensifies the reliance on objectification norms, which drives self-objectification as a psychological defense (H6).

## 2 Study 1: objectification cultural worldview and creatureliness of genders

The study 1 aimed to test two core assumptions of Terror Management Theory (TMT) in explaining self-objectification: First, TMT posits that cultural worldviews predominantly objectify women rather than men, leading to adherence to the cultural worldviews of (self-)objectification of women as a defense against mortality. Second, TMT suggests that women are perceived as having more salient creatureliness, making them stronger reminders of human mortality. By presenting equally salient biological features, this study examined whether men’s and women’s bodies differ in perceived creatureliness and whether any observed gender differences in self-objectification tendencies align with the cultural worldview of objectification.

This study and the following studies were conducted following the ethical standards of the institutional research committee and the Helsinki Declaration. Both studies were reviewed and approved by the Medical Ethics Committee of Northwest University (approval No. 221111001).

### 2.1 Method

#### 2.1.1 Participants and procedure

Participants were recruited using the online platform Questionnaire Star, with links distributed via social media groups of the research team members. A total of 112 valid responses were collected (*M*_age_ = 25.07 years, *SD* = 4.93), comprising 62 females (55.36%) and 50 males (44.64%).

Participants were informed that the study was a survey on sociocultural attitudes. After providing informed consent, participants completed measures assessing perceptions of objectification, cultural worldviews, and creatureliness for both male and female targets. The order of male/female versions for objectification and creatureliness measures was randomized, and filler questions were included to reduce carryover effects. While the convenience sample may limit generalizability, this approach provided an efficient initial test of our assumptions. Future studies could employ more diverse sampling strategies to broaden applicability.

#### 2.1.2 Measures

##### 2.1.2.1 Objectification cultural worldview

The Awareness Subscale of the Sociocultural Attitudes Toward Appearance Questionnaire (SATAQ) (Wang et al., 2019), adapted by Wang et al. for the Chinese context, was used to measure the objectification of females in sociocultural (Morris et al., 2014). In this study, the subject of each statement was modified to specifically refer to “men” or “women” (e.g., “*In our culture, it is important for a man/woman to be attractive to get more opportunities*”) to assess participants’ perceptions of objectification toward both genders in cultural worldview. The Cronbach’s *α* of the subscale was 0.836, indicating good reliability.

##### 2.1.2.2 Perceived creatureliness

We developed two parallel scales assessing how strongly participants associated male or female bodies with “animal-like” characteristics. The scale was based on previous TMT research, which related women’s creatureliness with reproductive characteristics such as “*prominent breasts*,” “*menstruation*,” and “*pregnancy*” (Eisler, 1995). To construct the male version, 10 graduate students in psychology provided open-ended responses, and the most representative features—“*prominent muscles*,” “d*ense body hair*,” and “*body odor*”—were selected. Both genders’ biological characteristics are encompassed within the key elements proposed by TMT that remind humans of their inevitable mortality: the body, its functions, and its byproducts (Goldenberg et al., 2000).

In the study 1, participants were asked to rate how these features made them perceive the individual as similar to an animal on a 7-point Likert scale (1 = not at all, 7 = very much). The Cronbach’s *α* for the male and female versions of the scale were 0.858 and 0.876, respectively, indicating high reliability. Although this approach reflects prior TMT research on creatureliness (Goldenberg et al., 2001; Beatson and Halloran, 2007), future studies may refine this measure by considering broader biological vulnerabilities (e.g., aging, illness) to avoid reinforcing cultural stereotypes.

### 2.2 Results

Paired-sample *t*-test results indicated that women were perceived as more culturally objectified.

(*M* = 5.02, *SD* = 0.82) than men (*M* = 4.63, *SD* = 0.96), *t* (111) = 3.40, *p* = 0.001. Male bodies were rated as exhibiting higher creatureliness (*M* = 5.50, *SD* = 1.11) than female bodies (*M* = 3.66, *SD* = 1.60), *t* (111) = −10.13, *p* < 0.001. The descriptive statistics are presented in Table 1.

**Table 1:** Objectification cultural worldview and creatureliness of genders.

Variables	Male		Female	
	*M*	*SD*	*M*	*SD*
Objectification cultural worldview	4.63	0.96	5.02	0.92
Creatureliness	5.50	1.11	3.66	1.60

### 2.3 Discussion

These findings offer a nuanced picture of how objectification culture and perceived creatureliness intersect with gender. Consistent with prior research (Fredrickson and Roberts, 1997; Calogero et al., 2011), women were seen as more heavily objectified, suggesting that cultural norms still emphasize female appearance as a key source of value. However, the notion that women’s bodies uniquely evoke creatureliness and remind individuals of mortality (Goldenberg et al., 2013) was not supported. Instead, men’s biological features were rated as more closely associated with “animal-like” qualities. This unexpected result implies that perceived creatureliness may not be inherently tied to women’s biology as previously assumed, challenging a core TMT-based explanation for why women are preferentially objectified (Goldenberg, 2013).

The discrepancy between higher female objectification and higher male creatureliness suggests that objectification may not stem solely from perceiving women as more biologically evocative of mortality. Instead, deeply ingrained cultural worldviews might shape the default tendency to objectify women independent of their perceived creatureliness. At the same time, our creatureliness measure may have tapped cultural stereotypes linking men to strength, ruggedness, or other “animalistic” attributes, which do not necessarily translate into mortality reminders in the same way that female reproductive functions are theorized to do (Goldenberg and Roberts, 2004).

These results underscore the need for more refined operationalizations of creatureliness that extend beyond gendered biological markers (Goldenberg et al., 2000; Goldenberg et al., 2006). Incorporating indicators of human vulnerability common to both sexes—such as physical fragility or aging—may better capture the essence of creatureliness as a mortality cue. Additionally, future research should explore whether cultural objectification norms operate in similar or distinct ways for men and women when broader aspects of biological vulnerability are considered. This line of inquiry will help clarify whether women’s privileged status in objectification culture is genuinely driven by their reproductive biology or if it reflects more deeply rooted cultural traditions that persist regardless of who is perceived as more creaturely.

Overall, the study 1 lays the groundwork for examining the interplay of cultural worldview defense and creatureliness suppression in driving self-objectification. By demonstrating that women remain more objectified despite men’s higher perceived creatureliness, these findings highlight the complexity of disentangling biological cues from cultural norms—a key challenge addressed in the subsequent studies.

## 3 Study 2: the effect of adherence to objectification cultural worldview on gender-specific self-objectification under mortality salience

Study 2 aimed to determine whether adherence to the objectification cultural worldview—operationalized as the internalization of objectifying cultural norms—moderates the relationship between mortality salience (MS) and self-objectification and whether this moderation differs by gender. Building on the study 1’s findings, which challenged the assumption that women inherently represent more substantial mortality reminders due to their creatureliness, this study tested the hypothesis that mortality threats would increase self-objectification in both men and women, contingent upon their adherence to the objectification worldview.

### 3.1 Methods

#### 3.1.1 Participants and procedure

One hundred sixty adults (50% female; *M*_age_ = 23.35 years, *SD* = 4.72) were recruited through an online survey platform. Balanced gender recruitment ensured equal representation of men and women, and all participants’ data were included in the final analysis. A power analysis confirmed that a sample size of 160 would provide sufficient statistical power (0.897) to detect moderate-to-large effects (*f* = 0.3) based on prior TMT research (Burke et al., 2010) at a significance level of 0.05. All participants were undergraduate or graduate students from various disciplines, and approximately 70% reported having middle-class socioeconomic status (SES). Participants provided informed consent and were informed that the study concerned contemporary popular culture.

During recruitment, participants completed the Internalization Subscale of SATAQ to assess their inherent adherence to objectification cultural worldviews. The formal experiment was conducted online via the survey platform 1 week later. In the formal experiment, participants were randomly assigned to either the mortality salience or control condition using the standard death reminder paradigm (Greenberg et al., 1986). Participants in the MS condition responded to the Chinese version of two open-ended questions about their own death, while those in the control condition answered similar questions about pain (Nu, 2013). Then, the Chinese version of the *Positive and Negative Affect Scale* (PANAS) (Watson et al., 1988; Qiu et al., 2008) served as a filler task. Finally, the participants completed the Chinese version of the *Self-Objectification Questionnaire* (SOQ; Noll and Fredrickson, 1998) before receiving monetary compensation. All participants passed attention checks and provided complete data.

#### 3.1.2 Materials and measures

##### 3.1.2.1 Adherence to objectification cultural worldview

We used the Internalization Subscale of the Chinese version of the Sociocultural Attitudes Toward Appearance Questionnaire (SATAQ) (Wang et al., 2019) to operationalize adherence to the objectification cultural worldview. This scale consists of 8 items, measuring the degree to which individuals have internalized cultural norms that equate physical attractiveness with social value (e.g., “*I view the body image portrayed in the media as my ideal*,” “*Seeing photos of people with ideal body shapes makes me wish my body looked like theirs.*”) Items were rated on a 7-point Likert scale, with higher scores indicating stronger internalization and thus greater adherence to the objectification worldview. In this study, Cronbach’s *α* = 0.76, indicating acceptable reliability.

##### 3.1.2.2 Mortality salience (MS)

The classic death reminder paradigm induced mortality salience (Merino et al., 2024). Participants in the mortality salience condition answered two open-ended questions about their death: “*Please describe your thoughts and feelings when thinking about your own death*” and “*Imagine what will happen to your body after death.*” The control group responded to similar questions, replacing the word “death” with “pain” (Nu, 2013). This approach is practical in numerous studies related to terror management research (Burke et al., 2010).

##### 3.1.2.3 Emotion measurement/delay task

The Positive and Negative Affect Scale (PANAS) (Qiu et al., 2008) was administered to measure participants’ emotional states following the mortality salience manipulation and as a delay task to introduce a temporal buffer before subsequent measures (Goldenberg et al., 2001). The Chinese version of the PANAS includes 20 items, with 10 assessing positive affect (e.g., “*excited*”) and 10 assessing negative affect (e.g., “*distressed*”). Participants rated the extent to which they were experiencing each emotion “at the present moment” on a 7-point Likert scale (1 = very slightly or not at all, 7 = extremely). The Cronbach’s *α* coefficients for this study’s positive and negative affect subscales were 0.89 and 0.81, respectively, indicating good reliability.

##### 3.1.2.4 Self-objectification

State-level self-objectification was measured using the Chinese version of the Self-Objectification Questionnaire (SOQ) (Noll and Fredrickson, 1998). The SOQ assesses individuals’ relative importance of appearance-based versus competence-based attributes in defining their physical self-concept. Participants were asked to rank a list of 10 attributes (e.g., *weight*) according to how much each impacts the person’s physical self-concept. The respondent ranks the attributes from most important (Goldenberg et al., 2005) to least important (0), and the respondent can only assign one attribute to each level of importance. For scoring, the 10 attributes are divided into two categories, either appearance-related attributes (e.g., *sex appeal*) or competence-related attributes (e.g., *health*). Next, the scores for the two types of attributes are summed, and the total competence score is subtracted from the total appearance score. Final scores can range from −25 to +25, with higher scores corresponding to higher levels of self-objectification. The SOQ has demonstrated robust concurrent validity in prior studies involving men and women (Hu et al., 2023).

### 3.2 Results

Variance analysis revealed no significant effects of mortality salience on positive or negative affect (all *p*s > 0.05), consistent with prior TMT findings that MS affects worldview adherence without altering immediate emotional states (Greenberg et al., 2003). The descriptive statistics are presented in Table 2.

**Table 2:** Effect of mortality salience on gender-specific self-objectification.

Gender	MS		Control	
	*M*	*SD*	*M*	*SD*
Female	5.90	10.65	−2.40	12.63
Male	−2.43	11.49	−8.28	11.82

#### 3.2.1 Self-objectification

**Figure 1:**
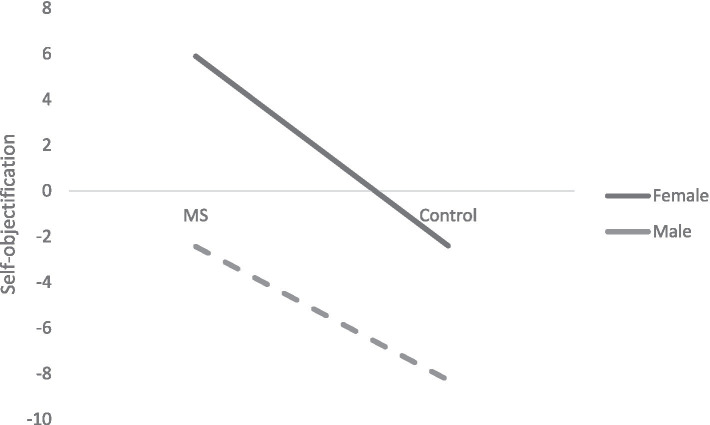
Influence of mortality salience a on self-objectification in males and females.

A two-way ANOVA (gender × MS) showed the main effects of gender and MS but no significant interaction. Women exhibited higher self-objectification overall, *F* (1, 156) = 14.81, *p* < 0.001, *ŋ*^2^ = 0.087. MS significantly increased self-objectification across both genders, *F* (1, 156) = 14.71, *p* < 0.001, *ŋ*^2^ = 0.086 (see Figure 1).

#### 3.2.2 Cultural worldview compliance and its moderating effect

A moderated moderation analysis (PROCESS Model 3) (Hayes, 2013) examined whether adherence to the objectification cultural worldview (operationalized via internalization scores), treating as a continuous variable, moderated the MS–self-objectification relationship and whether this effect differed by gender.

**Figure 2:**
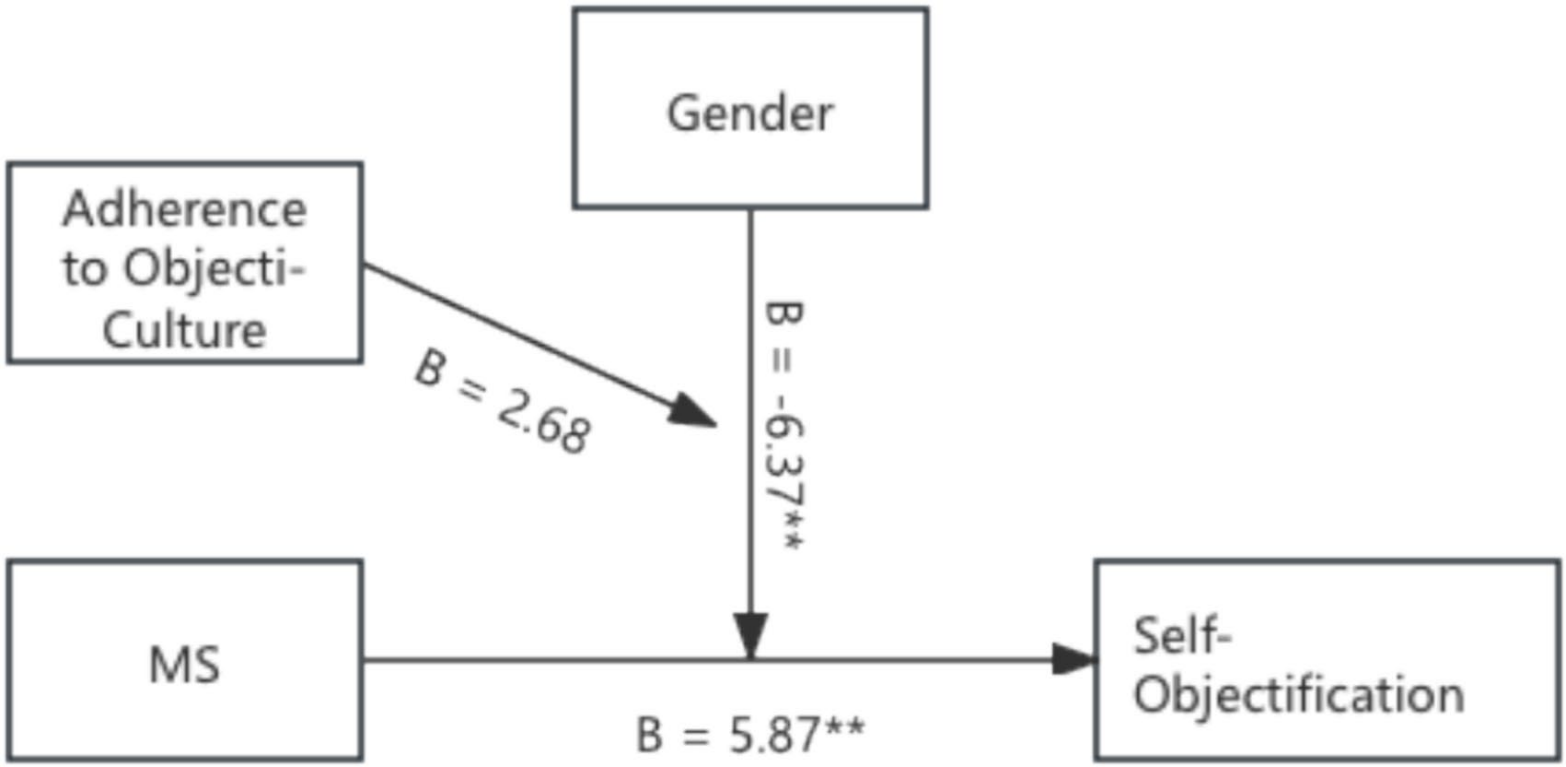
The moderated moderation model in Study 2. **p < 0.01.

The analysis revealed that MS significantly increased self-objectification (*B* = 5.88, *t* = 3.05, *p* = 0.003), indicating that individuals exposed to mortality reminders displayed higher self-objectification levels than those in the control condition. Gender had a significant main effect on self-objectification (*B* = −6.37, *t* = −3.31, *p* = 0.001), with females exhibiting significantly higher (*M* = 4.96, *SD* = 1.14) compared to men (*M* = 3.93, *SD* = 0.94), *F* (1, 158) = 38.77, *p* < 0.001, *ŋ*^2^ = 0.197. The three-way interaction (MS × gender × adherence) was insignificant (*p* = 0.113), indicating no gender-specific moderation pattern. However, the MS × adherence interaction approached significance (*B* = 2.69, *t* = 1.72, *p* = 0.08), suggesting a trend wherein individuals who more strongly internalized objectification norms (i.e., had higher adherence) showed a more pronounced self-objectification response to MS. Subsequent conditional analysis confirmed that at high adherence levels, MS robustly increased self-objectification (*B* = 10.63, *t* = 3.97, *p* < 0.001), but it was not significant at lower levels of adherence (*B* = 3.34, *t* = 1.25, *p* = 0.214) (see Figure 2).

### 3.3 Discussion

The results of Study 2 demonstrate that mortality salience (MS) increases self-objectification in both men and women, individuals’ adherence to the objectification cultural worldview—operationalized as their internalization of objectifying cultural norms—may influence this effect, although the interaction did not reach conventional significance levels. Although women displayed higher levels of self-objectification overall, men who strongly internalized these norms also showed increased self-objectification under MS. These preliminary findings suggest that objectification culture, as a part of the broader cultural worldview, may provide a means for both genders to cope with existential anxiety, though the effect did not reach conventional significance. In line with TMT, individuals confronted with their mortality appear motivated to affirm cultural standards that grant them symbolic value and continuity (Pyszczynski et al., 2004; Solomon et al., 2004).

Importantly, these findings must be considered alongside our study 1, which challenged the traditional TMT assumption that women’s bodies inherently provide more salient reminders of mortality due to their biological features (Goldenberg et al., 2013). While the study 1 indicated that gender differences in how men and women perceive objectification and biological vulnerability, we did not find substantial gender differences or interactions in self-objectification under the influence of mortality salience (MS). This suggests that, while cultural worldview compliance may explain some of the gendered aspects of self-objectification (i.e., women’s stronger internalization of objectifying cultural norms), functions more universally across genders. MS increased self-objectification in both men and women, with a trend suggesting that individuals with higher adherence to objectification norms may be more responsive to MS. This apparent discrepancy raises the possibility that objective biological cues do not solely determine the link between creatureliness and objectification. Instead, it may be shaped by cultural narratives that position women’s bodies as key targets of objectification (Hebl et al., 2004), regardless of whether they are objectively more creaturely.

From this perspective, individuals’ adherence to the objectification cultural worldview may reflect a more intense or nuanced form of “creatureliness suppression.” Traditional TMT explanations suggest that objectifying women’s bodies helps distance individuals from mortality-related thoughts tied to biology and reproduction (Goldenberg, 2013; Eisler, 1995). While the study 1’s results complicate the notion that women’s biology is the direct source of stronger creatureliness cues, the findings suggest that individuals who strongly internalize objectification norms, regardless of gender, may respond to existential threats by reinforcing these norms (Calogero et al., 2011), though the effect did not reach conventional significance. In other words, even if women’s bodies are not objectively more creaturely, cultural worldviews have long cast women’s physicality as symbolically vulnerable (Fredrickson and Roberts, 1997), thus motivating a stronger adherence to objectification as a strategy to downplay that vulnerability and minimize death anxiety.

This interpretation provides preliminary evidence that adherence to objectification norms may influence creatureliness suppression strategies, potentially varying based on the degree of internalization rather than gender per se (Hebl et al., 2004). For women, a cultural history of heightened objectification may have led to a more entrenched pathway: when mortality salience, turning to objectifying cultural norms offers a means to “de-creature” oneself, transforming biological realities into symbols of attractiveness or desirability. Even when perceived as more creaturely, men may not face the same cultural pressure to rely on objectification norms for existential buffering—unless, as our data show, they have strongly internalized these norms. Thus, adherence to the objectification worldview may function as a flexible coping mechanism for both genders, though this interpretation requires careful consideration given the marginal nature of the moderating effect. However, historically, women have been more consistently guided into this pathway due to cultural expectations.

These insights highlight the need to examine how creatureliness suppression and cultural worldview compliance interact. The absence of a three-way interaction does not negate the relevance of gender; instead, it emphasizes that gender differences in self-objectification under MS may stem more from longstanding cultural pressures and socialization processes than from inherent biological disparities. To clarify these relationships, it is essential to directly manipulate creatureliness cues and observe their interplay with mortality salience and adherence to objectification norms.

This necessity sets the stage for Study 3. By directly testing the hypothesized interplay between creatureliness and MS, we can determine whether making human vulnerability salient intensifies the reliance on objectification norms and whether this process is indeed more pronounced in women due to their historically entrenched cultural positioning. Such an investigation will shed light on whether adherence to objectification cultural worldviews serves as a universal existential defense mechanism—available to both genders when properly “primed” by cultural narratives and personal internalization—and how creatureliness suppression may operate as a distinct yet intersecting pathway to mitigating death anxiety.

## 4 Study 3: the effect of creatureliness salience on self-objectification under mortality salience

Study 3 employed a 2 (Mortality Salience: mortality salience/control) × 2 (Creatureliness Salience: heightened/reduced) × 2 (Gender: male/female) three-factor between-subjects design, with self-objectification as the dependent variable and adherence to objectification cultural worldview as the potential mediator.

### 4.1 Method

#### 4.1.1 Participants and procedure

A power analysis was conducted. We set the effect size *f* = 0.3 based on prior TMT studies. For a 2 × 2 × 2 factorial design with an *α* level of 0.05, a sample size of 165 was calculated to provide a power of 0.76. Although this is slightly below the recommended 0.8 threshold, it is within acceptable limits for exploratory studies. All participants were undergraduate or graduate students from various disciplines, reporting middle-class status, and 20% identified as lower-class.

Participants were informed that the study consisted of three independent parts: (1) an implicit personality test (mortality salience induction), (2) an evaluation of “Outstanding Student Essays,” and (3) a body cognition survey. Following informed consent, participants completed the mortality salience/control prompts, the PANAS scale, a randomly assigned essay task, the SOQ, and the SATAQ Internalization Subscale. Demographic information was collected at the end. All participants received monetary compensation upon completion. Ten participants from the classroom setting were interviewed afterward, and none indicated suspicion of the study’s true purpose.

#### 4.1.2 Materials and measures

##### 4.1.2.1 Mortality salience and delay task

Same as study 2.

##### 4.1.2.2 Creatureliness salience

Participants were randomly assigned to read one of two short essays adapted from Goldenberg et al. (2001). The essays manipulated awareness of creatureliness:

Heightened Creatureliness: Emphasizing human-animal similarities (e.g., “*Humans are also a kind of animal…*”)/ Reduced Creatureliness: Emphasizing human uniqueness (e.g., “*Humans are truly unique…*”).

Participants rated their reactions to the essays on six items (e.g., “*Do you like the author?*”) on a 7-point Likert scale, with the average score indicating participants’ reactions (Cronbach’s *α* = 0.73).

##### 4.1.2.3 Manipulation check

Two questions adapted from previous studies (Mountain and Marino, 2015) were used: “*To what extent do you think humans and animals are similar*?” and “*Which of the following distances best reflects the relationship between humans and animals*?” to assess the effectiveness of the creatureliness manipulation rated on a 7-point Likert scale, with the average score indicating participants’ perceived creatureliness (Cronbach’s *α* = 0.64).

##### 4.1.2.4 Self-objectification

The Chinese version of the SOQ from Study 2 was used.

##### 4.1.2.5 Adherence to objectification cultural worldview

The SATAQ Internalization Subscale from Study 2 was used.

### 4.2 Results

#### 4.2.1 Creatureliness salience manipulation check

An independent-sample *t*-test confirmed the manipulation’s effectiveness. Participants in the “Humans are Similar to Animals” condition reported higher perceived creatureliness (*M* = 4.78, *SD* = 1.37) than those in the “Humans are Unique” condition (*M* = 3.96, *SD* = 1.45), *t*(163) = −3.728, *p* < 0.001, Cohen’s *d* = 0.58. Although individuals under mortality salience tended to deny human-animal similarity, manipulating creatureliness was still effective in altering perceived creatureliness.

#### 4.2.2 Reaction to the essays

A 2 (mortality salience: mortality/control) × 2 (creatureliness salience: heightened/reduced) × 2 (gender: male/female) ANOVA revealed a significant main effect of creatureliness salience, *F*(1, 157) = 45.76, *p* < 0.001, *ŋ* ^2^_ = 0.226, with the “humans are unique” essay (reduced creatureliness salience) receiving higher ratings (*M* = 4.81, *SD* = 0.85) compared to the “humans similar to animals” essay (heightened creatureliness salience; *M* = 3.86, *SD* = 1.05). The result indicates that participants favored affirming human uniqueness over acknowledging creatureliness overall. No significant main or interaction effects involving gender were observed, *F*(1, 157) = 0.02, *p* = 0.879, indicating that gender did not influence the evaluations of the essays. These findings support that mortality salience leads individuals to affirm human uniqueness and reject creatureliness, consistent with terror management theory (Goldenberg et al., 2001; Mountain and Marino, 2015). The lack of gender differences suggests that this pattern is robust across genders.

#### 4.2.3 Self-objectification

**Figure 3:**
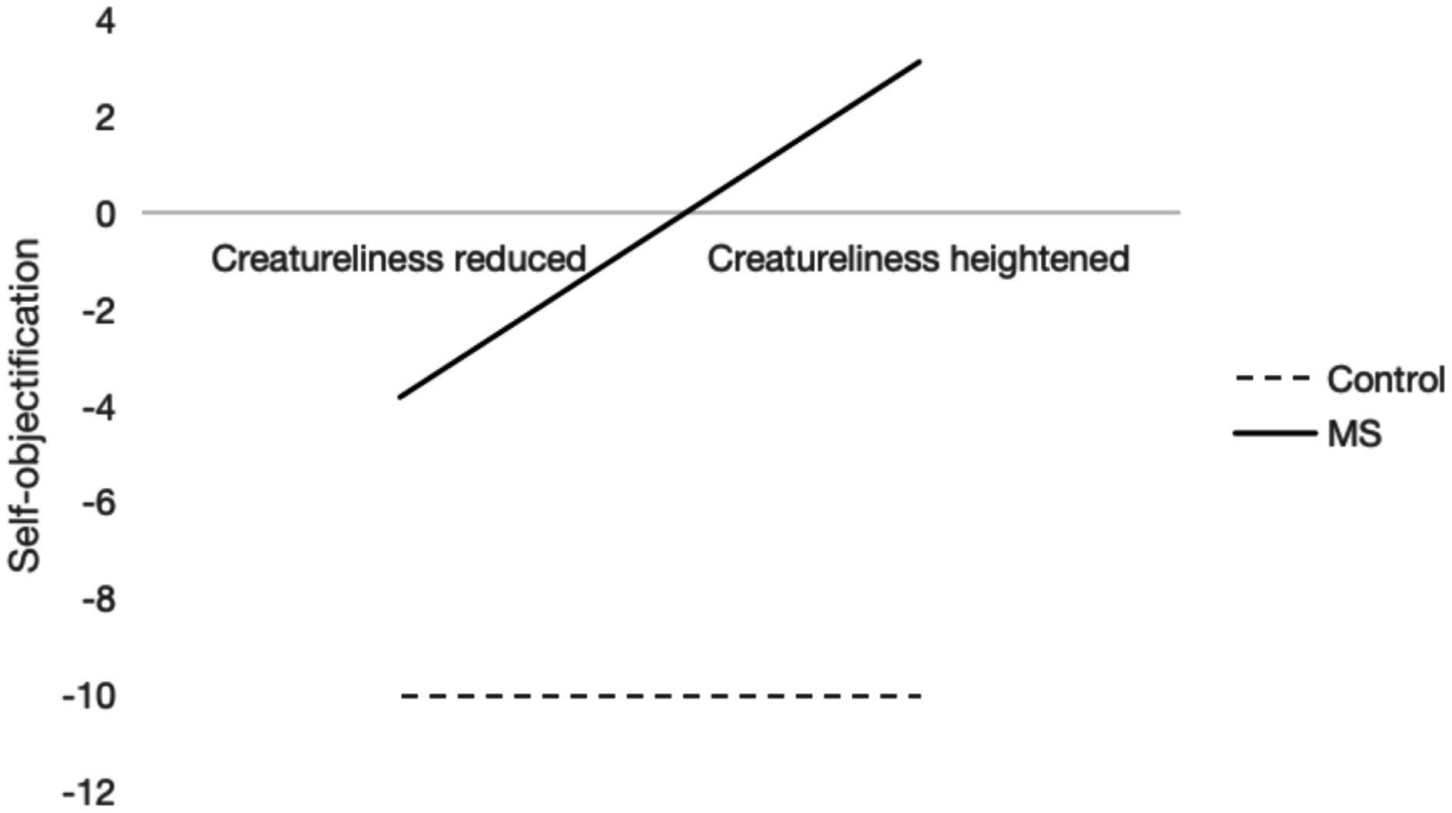
Influence of mortality salience and creatureliness on self-objectification.

A 2 (mortality salience: mortality/control) × 2 (creatureliness salience: heightened/reduced) × 2 (gender: male/female) ANOVA was conducted on self-objectification. The three-way interaction was insignificant, *F*(1, 157) = 0.597, *p* = 0.441. However, the main effects of mortality salience (*F*(1, 157) = 46.07, *p* < 0.001, *ŋ*^2^ = 0.227) and creatureliness salience (*F*(1, 157) = 5.95, *p* = 0.016, *ŋ*^2^ = 0.037) were significant, as was their interaction (*F*(1, 157) = 5.95, *p* = 0.016, *ŋ*^2^ = 0.037) (see Figure 3). Gender also had a significant main effect (*F*(1, 157) = 32.64, *p* < 0.001, *ŋ*^2^ = 0.172). Simple effect analysis revealed that, after mortality salience, heightened creatureliness significantly increased self-objectification, *t*(163) = 3.408, *p* = 0.001, Cohen’s *d* = 0.765, while for the control group, creatureliness salience did not affect self-objectification, *p* > 0.05. Descriptive statistics are presented in Table 3.

**Table 3:** Influence of mortality salience and creatureliness on self-objectification in males and females.

	Gender	Creatureliness heightened		Creatureliness reduced	
		*M*	*SD*	*M*	*SD*
MS	Female	5.82	9.36	1.52	6.91
	Male	6.56	9.24	−7.94	9.35
Control	Female	−5.61	9.82	−6.10	11.15
	Male	−3.35	8.97	−8.47	10.70

#### 4.2.4 Adherence to objectification cultural worldview

A 2 (mortality salience: mortality/control) × 2 (creatureliness salience: heightened/reduced) × 2 (gender: male/female) ANOVA was conducted on adherence to objectification cultural worldview. The three-way interaction was insignificant, *F*(1, 157) = 0.992, *p* = 0.321, either the interaction between mortality salience, creatureliness salience, and gender (*P*s > 0.5). The main effects of gender were significant, *F*(1, 157) = 6.824, *p* = 0.010, *ŋ*^2^ = 0.042; however, the main effects of mortality salience and creatureliness salience were insignificant. The results indicate that adherence to objectification culture was not significantly affected by MS or creatureliness salience, individually or in combination. Therefore, we did not further analyze the potential mediating role of adherence to objectification culture in inducing self-objectification.

### 4.3 Discussion

The findings of Study 3 extend our understanding of how mortality salience (MS) and creatureliness salience jointly influence self-objectification, building directly on the insights from the study 1 and Study 2. Consistent with TMT’s core propositions, we found that MS again heightened self-objectification (Goldenberg et al., 2000; Goldenberg et al., 2005). More importantly, participants exposed to heightened creatureliness salience under MS exhibited significantly greater self-objectification, which indicates that reminding individuals of their animalistic nature amplifies existential anxiety and prompts them to reaffirm appearance-based standards (Goldenberg and Morris, 2016; Grabe et al., 2005). This result aligns with the notion that creatureliness suppression—striving to distance oneself from biological reminders of mortality—serves as a key psychological defense mechanism (Heflick et al., 2011).

Notably, the absence of a three-way interaction (MS × creatureliness salience × gender) suggests that creatureliness suppression operates as a broadly applicable coping strategy rather than a gender-specific one. Both men and women showed increased self-objectification under MS when creatureliness was made salient, supporting the idea that existential concerns about human fragility can trigger objectification-related defenses across genders. While cultural worldview compliance (i.e., adherence to objectifying cultural norms) explains baseline gender differences in self-objectification, the acute response to explicit creatureliness cues appears more universally human.

The results also provide insight into a core question raised in earlier studies: Is women’s prominent role in objectification culture attributable to greater inherent creatureliness, or does it reflect deeper cultural processes? Although the study 1 challenged the notion that women’s bodies are objectively more creaturely reminders, Study 3 shows that both genders respond similarly when creatureliness cues are explicitly highlighted, which suggests that the prominence of women’s objectification in cultural worldviews may stem more from historically entrenched norms and stronger internalization of these norms by women, rather than from any objective, biological difference in mortality reminders. Earlier findings indicated that women, on average, internalize objectification norms more strongly (Hebl et al., 2004), thereby showing more pronounced self-objectification under MS. Study 3’s results clarify that when creatureliness is salient, existential anxiety is universally felt. Both genders turn to objectification as a defense. However, since women generally have higher adherence to objectification cultural worldview, they may still display a somewhat more robust response—though the current study’s non-significant three-way interaction suggests that the potency of creatureliness suppression under MS is not exclusively tied to gender.

Although adherence to objectification cultural norms did not significantly change as a function of Mortality Salience (MS) or Creatureliness Salience in this study, this finding may be attributed to the insensitivity of the measurement scale to the experimental manipulations. Compared to the measurement of Self-Objectification, the adherence scale may be more susceptible to social desirability bias, which could account for the lack of significant effects observed. Social desirability bias refers to the tendency of participants to respond in a manner that they perceive as socially acceptable or desirable, rather than reflecting their true attitudes or behaviors. This is especially likely in measures related to cultural norms, where individuals might underreport or overreport their adherence to objectifying standards due to concerns about how they are perceived by others.

However, the previous findings indicate that such adherence remains a critical baseline factor influencing how strongly self-objectification emerges. The current results suggest that once existential anxiety and creatureliness cues are activated, the immediate turn to objectification as a defense mechanism, bypassing the need for further shifts in the internalization of cultural norms. In other words, the activation of existential anxiety may drive individuals to rely on their pre-existing adherence to objectification norms, applying them more intensively when confronted with reminders of mortality and biological vulnerability. This response indicates that self-objectification may be more of an immediate, reactive defense mechanism, rather than one that requires the modification of cultural beliefs or internalization processes.

Given the central role of baseline adherence to objectification norms, future studies might consider refining the measurement tools to better capture shifts in adherence to objectification culture, ensuring that such measurements are more sensitive to experimental manipulations. Additionally, future research should explore whether other factors, such as individual differences in susceptibility to social influence or varying levels of cultural identification, might affect the relationship between MS, creatureliness salience, and adherence to objectification norms.

## 5 General discussion

This research aimed to clarify how self-objectification is a defense against death anxiety by examining cultural worldview compliance and creatureliness suppression within a Terror Management Theory (TMT) framework. Study 1 tested foundational assumptions, revealing that while women’s bodies are culturally more objectified, men were perceived as more “creaturely,” calling into question simplistic biological explanations for female objectification. Building on this, Study 2 manipulated mortality salience (MS) and measured adherence to objectification norms, finding that both men and women who internalized these norms were more likely to self-objectify under MS, suggesting that objectification may function as a gender-transcendent existential buffer. Study 3 introduced explicit creatureliness cues alongside MS, demonstrating that emphasizing human-animal similarity heightened self-objectification in both sexes, independently of immediate cultural norm adherence shifts. Together, these studies refine TMT’s understanding of objectification culture, indicating that their biology does not inherently drive women’s prominence in objectification but reflects cultural constructions and flexible defense mechanisms that both sexes may employ when facing mortality reminders.

### 5.1 Interpretation and implications

These results suggest a more intricate interplay of cultural and biological factors than previously assumed. Rather than relying on the premise that women’s bodies are objectively more creaturely and thus more readily objectified, our findings imply that women’s prominent role in objectification culture may stem mainly from the more profound and more pervasive internalization of objectification norms historically imposed on them (Siegel and Calogero, 2019; Schaefer et al., 2017). In other words, cultural narratives and power structures—rather than immutable biological facts—shape who is objectified and who engages in self-objectification when confronted with mortality. While feminist and socio-cultural perspectives have long argued that female objectification arises from unequal power relations (Moya-Garófano et al., 2021), our research integrates TMT with these viewpoints, showing that attributing objectification solely to women’s biology not only oversimplifies the phenomenon but also risks reinforcing existing gender biases (Perach, 2019; Sreekanth, 2019).

By distinguishing the roles of cultural worldview compliance and creatureliness suppression, this research provides a more balanced and comprehensive picture of how MS leads individuals to engage in self-objectification. Previous studies often conflated these mechanisms by focusing on women’s reproductive features, potentially overlooking how men might also resort to self-objectification under specific existential pressures. Our approach, which used general human-animal similarity cues, suggests that creatureliness suppression can trigger objectification across genders. Understanding this interplay may open the door to more nuanced and effective ways of addressing the psychological costs of objectification, emphasizing cultural transformation over biological determinism, and ultimately promoting wellbeing for all.

### 5.2 Significance and contributions

This research offers a more comprehensive and flexible conceptual framework for understanding self-objectification within the Terror Management Theory (TMT) paradigm, moving beyond traditional gender-based and biologically driven explanations. While the preceding Interpretation and Implications section detailed how cultural worldview adherence and creatureliness suppression independently and jointly shape self-objectification under mortality salience (MS), here we focus on these insights’ broader theoretical and practical significance.

Theoretically, our findings integrate feminist and social constructionist perspectives into TMT, suggesting that objectification may serve as a universal existential defense rather than a uniquely female vulnerability. We enrich TMT’s explanatory scope by highlighting that both men and women may engage in self-objectification when cultural norms or biological cues are salient. This expanded model underscores that objectification culture is not simply a byproduct of women’s supposed inherent biology but may be a malleable, culturally constructed phenomenon. Such a perspective encourages future research to explore how varying social, historical, and cultural contexts influence who internalizes objectification norms and how they respond to existential threats.

From a practical standpoint, our findings underscore the importance of focusing interventions on cultural and social factors. Since women’s higher self-objectification in non-creatureliness contexts may be primarily due to their deeper internalization of objectification norms, efforts to reduce the psychological harm of self-objectification should target these cultural narratives and values. Interventions aimed at questioning and reshaping the cultural emphasis on female appearance may alleviate women’s vulnerability under mortality threats. This broader perspective aligns more closely with feminist and social constructivist approaches, suggesting that interventions should target cultural and social conditions to reduce the prevalence and harm of objectification (Hassan et al., 2023; Feltman and Szymanski, 2018; de Wide et al., 2020). Similarly, acknowledging that men can turn to self-objectification when creatureliness is salient suggests that reducing reliance on appearance-based standards for both genders may help mitigate existential anxiety. Interventions could involve promoting attributes unrelated to physical appearance and providing alternative forms of meaning, such as emphasizing personal achievements, prosocial behaviors, or communal connections (Shi et al., 2022; Wang and Ding, 2023; Calkins et al., 2023).

### 5.3 Limitations and future direction

Despite this research’s theoretical and empirical contributions, several limitations warrant attention. First, our operationalization of self-objectification focused solely on appearance-related attributes, leaving other dimensions—such as sexual objectification, dehumanization, body surveillance, and body shame (Moradi and Huang, 2008)—unexamined, which constrained our ability to fully capture the complexity of self-objectification and its nuanced relationship with mortality salience (MS). We are designing follow-up studies incorporating more diverse measurement tools, including scales targeting sexual objectification and experiential indicators (e.g., reduced flow), to better differentiate how cultural worldview compliance and creatureliness suppression manifest across various self-objectification domains.

Second, while we identified the roles of cultural worldview adherence and creatureliness suppression as distinct yet interacting mechanisms, we did not systematically manipulate both defenses within the same experimental design, which leaves open questions about their relative priority, compensatory functions, or potential conflict. For instance, it remains to explore how individuals might respond when objectifying cultural norms are not aligned with or contradicting creatureliness suppression strategies. Future experimental work could concurrently prime these two pathways to clarify their dynamic interplay. Although we have initiated preliminary discussions and pilot testing, disentangling these processes requires more complex study designs that we have yet to implement.

Third, the cultural specificity of our findings must be considered. We examined our hypotheses within a contemporary Chinese context, where male objectification norms have historically been less salient than those for women. However, social changes and Western cultural influences may increase pressures on men’s physical appearance (Thornborrow et al., 2020; Grabe et al., 2008). Whether our findings generalize to other cultural milieus or contexts where male objectification is more entrenched remains an open question. We plan to extend our research cross-culturally, investigating different societies and historical moments to understand how evolving objectification norms shape existential defenses. Longitudinal studies could track how shifts in cultural standards influence the terror management functions of self-objectification for men and women over time.

Finally, our reliance on convenience sampling may limit the generalizability of the results. Although this approach facilitated efficient data collection, it may have introduced sampling biases. In future studies, we intend to adopt more systematic sampling strategies—such as stratified random sampling or panel surveys—to ensure that our findings hold across diverse demographic and psychological profiles. Moreover, ideal research might encompass multi-site collaborations, representative samples, and longer-term interventions that more robustly test the robustness and scope of our theoretical model.

## 6 Conclusion

This research investigated how mortality salience influences self-objectification through cultural worldview compliance and creatureliness suppression, offering a more nuanced and flexible account than traditional gender-based explanations. Our integrated findings reveal that women’s heightened self-objectification primarily reflects deeper cultural internalization of objectification norms rather than any inherent biological vulnerability. At the same time, men may also resort to self-objectification when creatureliness cues make their shared human fragility more salient. These results highlight that objectification may serve as a universal, context-dependent existential defense rather than a phenomenon tied exclusively to female biology or cultural pressures on women.

By bridging TMT with feminist and social constructionist perspectives, we demonstrate that existential defenses can unfold along multiple pathways, influenced by shifting cultural narratives and biological reminders. This research expanded theoretical framework encourages future research to consider how evolving social norms, historical changes, and cross-cultural variations shape self-objectification patterns. Ultimately, understanding this interplay can inform interventions to reduce objectification’s psychological and social costs, emphasizing cultural transformation and broader social values as key routes toward enhancing overall wellbeing.

## Data Availability

The raw data supporting the conclusions of this article will be made available by the authors without undue reservation.
